# Borderline personality disorder in Irish Travellers: a cross-sectional study of an ultra-high-risk group

**DOI:** 10.1192/j.eurpsy.2023.227

**Published:** 2023-07-19

**Authors:** K. Tong, S. Costello, E. McCabe, A. M. Doherty

**Affiliations:** 1 National Forensic Mental Health Service, Central Mental Hospital, Dundrum, Dublin; 2 Department of Psychiatry, University Hospital Galway, Galway; 3 Department of Psychiatry, School of Medicine & Medical Science, University College Dublin; 4 Department of Psychiatry, Mater Misericordiae University Hospital, Dublin, Ireland

## Abstract

**Introduction:**

Irish Travellers are recognised as a minority ethnic group in Ireland. While mental health services are available to Travellers, these services are often perceived as inadequate at addressing the mental health needs of this population. Studies have shown that there is a higher prevalence of mental disorders in the Traveller community in Ireland compared to the general Irish public. However, it is unclear if Travellers are at a higher risk of developing personality disorders, particularly borderline personality disorder.

**Objectives:**

This study will examine the prevalence of borderline personality disorder and other mental disorders in Travellers attending a community mental health service in Tuam, County Galway. This study will also investigate the biopsychosocial interventions delivered to this cohort and the clinical outcome following the interventions.

**Methods:**

This is a cross-sectional study. Travellers who were active caseloads on the register of the community mental health service in Tuam were included in this study. Chart reviews were carried out on all samples included in this study.

**Results:**

A total of 59 active patients were included in this study. The Traveller community formed 14.4% (59 out of 410) of the active caseloads of the Tuam mental health service. There were more male than female Travellers who attended the service. Mean age was 36 years old. The most common mental disorder in this study cohort is depressive episode (F32). This is followed by mixed anxiety and depressive disorder (F41.2). A significant minority (9, 15.3%) of the study participants were given a diagnosis of borderline personality disorder. 9 study participants await diagnostic clarification. Nearly one-fifth (18%, n=9) of the study participants with a diagnosis had been given a diagnosis of borderline personality disorder. Over 50% of the study participants were on at least 3 different medications from at least 2 different classes of psychotropics.

**Conclusions:**

This study shows that there is a significant overrepresentation of Travellers attending the community mental health service in Tuam. The findings from this study can be used to plan future service development projects to better meet the needs of this unique population.

**Disclosure of Interest:**

None Declared

